# A probabilistic model to evaluate the effectiveness of main solutions to COVID-19 spreading in university buildings according to proximity and time-based consolidated criteria

**DOI:** 10.1007/s12273-021-0770-2

**Published:** 2021-02-27

**Authors:** Marco D’Orazio, Gabriele Bernardini, Enrico Quagliarini

**Affiliations:** Department of Construction, Civil Engineering and Architecture, Università Politecnica delle Marche, via Brecce Bianche, 60131 Ancona, Italy

**Keywords:** simulation model, closed built environment, building occupancy, crowd models, proximity exposure, COVID-19

## Abstract

University buildings are one of the most relevant closed environments in which the COVID-19 event clearly pointed out stakeholders’ needs toward safety issues, especially because of the possibility of day-to-day presences of the same users (i.e. students, teachers) and overcrowding causing long-lasting contacts with possible “infectors”. While waiting for the vaccine, as for other public buildings, policy-makers’ measures to limit virus outbreaks combine individual’s strategies (facial masks), occupants’ capacity and access control. But, up to now, no easy-to-apply tools are available for assessing the punctual effectiveness of such measures. To fill this gap, this work proposes a quick and probabilistic simulation model based on consolidated proximity and exposure-time-based rules for virus transmission confirmed by international health organizations. The building occupancy is defined according to university scheduling, identifying the main “attraction areas” in the building (classrooms, break-areas). Scenarios are defined in terms of occupants’ densities and the above-mentioned mitigation strategies. The model is calibrated on experimental data and applied to a relevant university building. Results demonstrate the model capabilities. In particular, it underlines that if such strategies are not combined, the virus spreading can be limited by only using high protection respiratory devices (i.e. FFP3) by almost every occupant. On the contrary, the combination between access control and building capacity limitation can lead to the adoption of lighter protective devices (i.e. surgical masks), thus improving the feasibility, users’ comfort and favorable reception. Simplified rules to combine acceptable mask filters-occupants’ density are thus provided to help stakeholders in organizing users’ presences in the building during the pandemic.

**Electronic Supplementary Material (ESM):**

supplementary material is available in the online version of this article at 10.1007/s12273-021-0770-2.

## Keywords simulation model;
closed built environment;
building occupancy;
crowd models;
proximity exposure;
COVID-19

## Article History

Indoor/Outdoor Airflow and Air Quality

## 1 Introduction

Buildings open to the public are critical scenarios for the occupants' safety in a pandemic (Ronchi and Lovreglio 2020), especially when crowd conditions occur (Dong et al. 2018). The COVID-19 pandemic evidences how the long-lasting presence of people in closed built environments increases the pandemic spreading (Cirrincione et al. 2020; Ronchi and Lovreglio 2020; Wang et al. 2020; Zizzo et al. 2020), as also demonstrated by real-world cases of closed environments (see, e.g., the Diamond Princess cruise emergency) (Fang et al. 2020; Mizumoto and Chowell 2020). Among the different exposure models (Ronchi and Lovreglio 2020), proximity and exposure-time-based factors of such a virus transmission seem to be the most relevant roles in respect to high-risk exposure/close contact definition, according to consolidate criteria of national and international health organizations[1] (Anderson et al. 2020; Bruinen de Bruin et al. 2020; Prem et al. 2020; Yang et al. 2020). Such basic rules consider that a probable case is a person remaining "in a closed environment

[1] e.g.: http://www.salute.gov.it/portale/nuovocoronavirus/dettaglioFaqNuovoCoronavirus.jsp?lingua=italiano&id=228, https://www.ecdc.europa.eu/en/covid-19/surveillance/surveillance-definitions (last access: 5/8/2020)

(e.g. classroom, meeting room, hospital waiting room, etc.) with a COVID-19 case for 15 minutes or more and at a distance of less than 2 m", whose conditions include any direct and indirect contact according to a proximity-based standpoint (Cirrincione et al. 2020; Zizzo et al. 2020).

In this general context, university buildings were (and still are) one of the most critical scenarios for the contagion spreading because of the exposure-increasing consequences due to the long-lasting presence (day-to-day) of the same users (i.e. students, teachers) (Anderson et al. 2020; Prem et al. 2020; Yang et al. 2020; Bruinen de Bruin et al. 2020): (1) in the same "*attraction areas*" hosting main Learning and Teaching (L&T) activities (classrooms) or breaks time (break areas); (2) in possible overcrowding conditions over time (e.g. due to the contemporary presence of the users in the main areas). As for other public buildings (Anderson et al. 2020; Bruinen de Bruin et al. 2020), the adoption of compulsory and widespread lockdowns of university buildings to limit the occupants' exposure caused and causes academic disruption and negative experiences of remote access to L&T by students (Dohaney et al. 2020; Favale et al. 2020).

Decision-makers moved towards the combination of more feasible mitigation strategies (Chen et al. 2020; Cirrincione et al. 2020; Fang et al. 2020; Howard et al. 2020; Murray et al. 2020; Prem et al. 2020; Servick 2020; Wilder-Smith et al. 2020; Yang et al. 2020)[1]: (a) social distancing solutions, which increase the possibility of maintaining safety distances (i.e. ranging from 1 m to 2 m depending on National Guidelines) by limiting the building occupants' capacity; (b) the use of respiratory protective devices (facial masks); (c) the control of access to the building by COVID-19 infectors, by using techniques for symptoms detection (i.e. fever). Nevertheless, they should understand the effective impact of each measure (and of their combination) to effectively set up acceptable solutions from the perspective of both the stakeholders (e.g. which solutions can be easily implemented with an effective impact on the activity?) and the final users (e.g. which solutions will lead to the possibility of restoring the "normal" fruition of the spaces?) (Romero et al. 2020; Ronchi and Lovreglio 2020). Simulation tools could be useful to this end, as also remarked by previous studies on airborne diseases mitigation (Saari et al. 2006; Gao et al. 2008; Laskowski et al. 2011; Gao et al. 2016; Zhang et al. 2018), as well as by other individuals' emergency safety applications in the built environment, including crowd conditions (Zheng et al. 2009; Casareale et al. 2017; Ronchi and Lovreglio 2020).

Although different tools based on experimental data have been developed to simulate COVID19 spreading at a wide-scale (e.g. national scale) and the impact of risk-mitigation strategies (Lopez and Rodo 2020; Prem et al. 2020; Fanelli and Piazza 2020), limited efforts have been performed for closed environment contexts (Mizumoto and Chowell 2020; Fang et al. 2020; Ronchi and Lovreglio 2020).

Thus, this study provides a simulation model to estimate the effectiveness of virus mitigation-measures to be adopted in university buildings. According to a quick-to-apply approach for building decision-makers' support tool development, the modelling methodology adopts a probabilistic approach to the virus transmission based on statistics (in view of Susceptible-Infected models) (Banos et al. 2015) to merge virus spreading and individuals' (students and teachers') occupancy of the building spaces. The main modelling criteria are based on the proximity and exposure-time-based factors as priority elements for virus spreading due to high-risk exposure/close contacts as stated above[1] (Fang et al. 2020; Ronchi and Lovreglio 2020; Yang et al. 2020) and according to previous works on airborne diseases transmission and simulation (Gao et al. 2016; Zhang et al. 2018; Prem et al. 2020). The occupants' rules are characterized in terms of presences in the different "*attraction areas*" (classrooms and break areas) depending on the L&T scheduling, and spreading-affecting additional features (i.e. occupants wearing a facial mask, being at a particular moment of the incubation period, being asymptomatic or not) (Mizumoto and Chowell 2020; Fang et al. 2020). The probabilistic approach enables to create different input scenarios and to evaluate possible differences in the spreading trends from a statistical point of view.

A real-world case study (the "Diamond Princess cruise") is used to calibrate the model and fit the contagion spreading data, thus reducing the gaps due to additional effects of secondary transmission ways (Azimi et al. 2020; Mizumoto and Chowell 2020). Finally, the calibrated model is applied to a significant university case study. The model capabilities are evaluated with respect to different mitigation strategies by providing insights on reliable solution combinations for decision-makers.

## 2 Model description and methods

### 2.1 Modelling approach

The spreading of COVID-19 between the occupants into the closed environment of university buildings is modelled by jointly representing the epidemic-related issues and the occupancy of the building spaces considered as "*attraction areas*" for the users during L&T activities (i.e. classrooms, break areas) (Banos et al. 2015; Fang et al. 2020). To this end, a probabilistic (statistics-based) approach to virus transmission and occupancy tasks is adopted, using ABM techniques and tools to assign effective rules to the agent, over space and time, including their spreading-affecting features (Laskowski et al. 2011; D'Orazio et al. 2014; Banos et al. 2015).

Concerning epidemic-related issues, the consolidated *proximity and exposure-time-based rules* representing high-risk exposure/close contacts are considered (Fang et al. 2020; Ronchi and Lovreglio 2020; Yang et al. 2020)[1]. Different transmission modes can be ideally considered, due to the presence of individuals in the same room/building at the same time (e.g. aerosol transmission route) (Azimi et al. 2020; Dai and Zhao 2020), the occupants' actions and space use (e.g. surface contamination) (Cirrincione et al. 2020; Hu et al. 2021; Prussin et al. 2020), as well as indoor (building-related) and outdoor (environmental aspects of the location where the building is placed) conditions (Adams et al. 2016; Lu et al. 2016, 2020). Nevertheless, we can assume that:

- to the date this paper was written (July 2020), they were not included in the main rules for international health organizations.
- parameters to clearly evaluate their impact are still under development and validation (e.g. in view of applying Wells–Riley equation).
- the infection probability due to aerosols seems to be limited to less than the 2% in case of effective control of Indoor Air Quality thanking to good ventilation systems (Dai and Zhao 2020), also in view of previous works on sick building syndromes (Lu et al. 2016).
- behavioral issues can impact the virus transmission, by including the role of hands contaminations due to contacts with surfaces, such as door openers or furniture (Cirrincione et al. 2020; Hu et al. 2021). Nevertheless, the overall influence of such transmission modes can be generally limited in view of: (1) current trends in risk awareness of both building stakeholders and population (Shiina et al. 2020; Knowles and Olatunji 2021); (2) the adoption of current international, national[2] and university regulations and guidelines requiring that people sanitize their hands when entering the building and each classroom (Cirrincione et al. 2020).

A general schematization of the overall epidemiological model approach is resumed in Figure 1, while Table 1 resumes the model parameters discussed below. Each simulated agent is characterized by:

- being infected or not. This status can vary at each step of the simulation. When the simulation starts, there is a certain percentage of occupants that is infected inside the built environment. The contagion timing is $t^*$ [simulation step]. At $t^* = 0$, a certain agent becomes infected. A "*delay*" period is considered as a latent period for infected agents, which corresponds to the initial phase during which the virus load increases up to provoke secondary infections (Lauer et al. 2020). $I_{delay}(t^*)$ [simulation step] is defined as the current step from the virus contagion. Once an individual is infected, $I_{delay}(t^*)$ increases at each simulation step by a *formcoeff* ($I_{delay}(t^*) = I_{delay}(t^*-1)+formcoeff$; in case of linear relationship, *formcoeff* = 1). Hence, *formcoeff* expresses the speed of the process after the moment of the contagion.
- wearing or not a respiratory protective device (in the following: *mask*). The percentage of people wearing the *mask*, as well as the *mask* filtering capacity (defined according to European standard EN 149:2001), could be decided in the setup of the model.
- being asymptomatic or not after being infected. In particular, not asymptomatic people are considered to "die" (leave/not enter the building) when the current step from the virus contagion $I_{step}$ [simulation step] reaches the timing to symptoms (e.g. fever) onset $I_{fev}$ [simulation step] (Lauer et al. 2020). The agent's "die" behaviour could both represent the effects of the disease for: (1) people who can be blocked by building access control system; (2) people who spontaneously leave/not enter the building due to their health conditions.

At each simulation step, the model evaluates if an *effective infector* is placed near uninfected ones, assuming a radius of 2 m as base proximity distance for contagion spreading *Dvir* [m][1]. In these conditions, each of the *effective infector* *i* has a chance to infect a neighbouring uninfected agent *j* according to Eq. (1), which is based on previous modelling approaches (Fang et al. 2020):

$$Pvir = \min\left(1, i_j \cdot t_{\exp} \cdot (1 - prot_i) \cdot (1 - prot_j) \cdot p_{\text{imm}}\right) \quad (1)$$

where:

- $i_j$ [—] is the transmission efficiency of the virus in the surrounding individual *j*, as in Eq. (2). Simple linearity between the incubation-related inputs is assumed.

$$i_j = \min\left(1, \frac{I_{\text{step}}}{I_{\text{inc}}}\right) \quad (2)$$

- $t_{\exp}$ [—] is the normalized exposure time of *i* in respect of *j* as in Eq. (3). It depends on the exposure time $\Delta t$ [simulation step] and the minimum contact time $t_c$ [simulation step] provoking the virus spreading. $t_c$ can be defined as the number of steps to simulate an equivalent time of 15 minutes. Simple linearity between the time-dependent inputs is assumed.

$$t_{\exp} = \min\left(1, \frac{\Delta t}{t_c}\right) \quad (3)$$

- $prot_i$ and $prot_j$ [—] are the level of protection given by the *mask filter* of the facial *masks* worn by the two agents.

[2] e.g. https://bit.ly/3aVqjDO (last access: 30/12/2020)

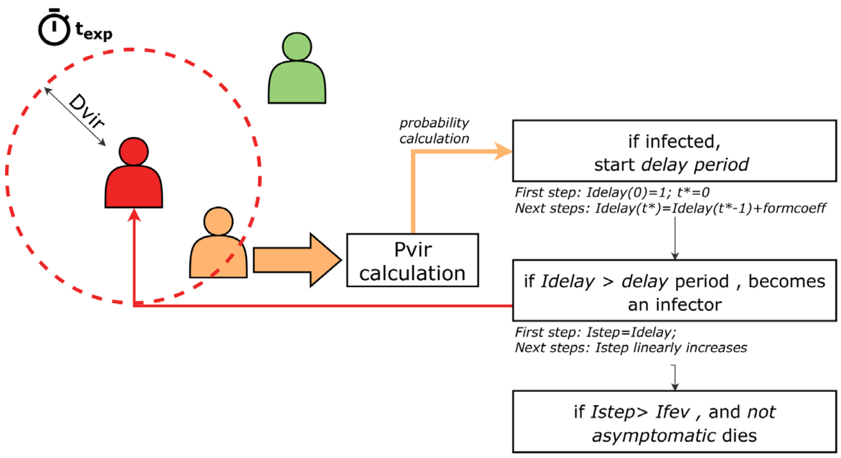

**Fig. 1** General scheme for the contagion spreading: the red agent is an infector, the green agent is out of *Dvir*, the orange agent is a possible infected agent. The *Pvir* calculation also depends on the exposure timing $t_{exp}$

**Table 1** Model parameters characterization

| Parameter | Unit of measure | Values range | Description |
|---|---|---|---|
| *N* | pp | > 0 | Number of people in the *world* |
| *A* | $m^2$, patches | > 0 | The dimension of the *world* in which the agents move |
| *formcoeff* | — | > 0 | Speed of the virus spreading, by modifying the delay increase for each step |
| $p_{imm}$ | % | 0 to 100 | If equal to 100%, all the population is virus-resistant (e.g. due to vaccine) |
| *Initial infectors %* | % | 0 to 100 | How many individuals could be infectors at the starting of the simulation |
| Asymptomatic ratio | % | 0 to 100 | How many individuals could be infectors without symptoms, thus not "dying" |
| Average delay | Simulation steps | > 0 | The average delay between being an infected individual and an effective infector, due to virus replication dynamics (Lauer et al. 2020). It also depends on how many steps represent each simulation day |
| $I_{inc}$ | Simulation step | > 0 | This is the incubation time, which starts from the contagion moment to the maximum considered display symptoms time (e.g. fever onset) for all the considered population (Lauer et al. 2020) |
| $I_{fev}$ | Simulation step | ≥ 0 | This is the time from the contagion to the minimum onset of the fever (compare to $I_{inc}$) (Lauer et al. 2020). In this work, the value is stochastically assigned within 0 to $I_{inc}$ |
| $prot_i$, $prot_j$ (*mask filter*) | — | 0 to 1 | Specific values can be assigned for respiratory protective devices categories for filtering half masks given by EN 149:2009 by considering maximum aerosol drops penetration percentage. Single mask characterization ranges are considered to include superior limits for each kind of mask: FFP3 ≥ 98%, 98% > FFP2 ≥ 95%; 95% > FFP1 ≥ 80%<br>Besides, previous works tried to classify surgical mask efficiency according to the NIOSH NaCl method (Rengasamy et al. 2017), by providing an efficiency range from 54% to 88%. Finally, a no-protection limit for *mask filter* from 0 to 25% is selected to consider the non-standards protection solutions, basing on the first quartile in uniform input distribution |
| $t_c$ | Simulation step | 1 step = 15 minutes | Number of steps to simulate an equivalent time of 15 minutes, according to consolidated data about indoor contagion spreading from national and international health organization[1] |
| *Mask wearing %* | % | 0 to 100 | Percentage of people implementing the considered protection level $prot_i$ |
| *Traveled distance* | Patches | ≥1 | Maximum distance between the previous and the next occupancy task for each agent, that is considered between two different simulation steps. It can depend on the distance between the areas in which the individual spends time. The maximum distance could be ideally set at $A^{1/2}$ by considering a squared *A* in the patches description |

The parameters vary from 0, which implies "no mask", to 1, which corresponds to maximum protection level. More data on the *mask filter* values adopted by this study is provided in Table 1.

- $p_{imm}$ [—] considers that "some people may be immune to the virus" (Fang et al. 2020), and it can vary from 0 to 1 (e.g., 0 corresponds to the case in which the whole population is vaccinated).

In Eq. (1), the adoption of the term 1 as a comparison factor allows limiting the upper probability *Pvir* to 100%. Once *Pvir* has been calculated, a random number (varying from 0 to 1) is compared to *Pvir* to stochastically define *j* as a new infected agent when the random number is lower or equal than *Pvir*. Once the agent has become infected, he/she will change its status into *effective infector* when the *delay* value will be reached.

Concerning the occupancy of the building spaces, the model considers that the agents are placed into a "close" *world*: there are no births and travel into or out of the simulated population, while deaths can exist because of the virus effects on the individuals. The model is limited to the fact that: (1) new infected can essentially occur inside the *world*; (2) infected people can remain inside until to "die". The model could consider that the same daily users frequent the university building (Gao et al. 2016; Zhang et al. 2018). Thus, they can return home during the evening, but the following day they will share the building spaces with the other same users. *N* [pp] is the number of simulated agents into the *world* and depends on the specific scenarios to be simulated. The *world* is characterized by "*attraction areas*" where the simulated agents can spend time according to the L&T activities schedule (classrooms and break areas). The classrooms are considered as the initial position where agents are generated, by considering an initial distance among the individuals randomly ranging from 1 m to 3 m. This assumption allows initially respecting social distancing. Agents will spend most of the time in classrooms, having the possibility of leaving their initial position at the lessons breaks. In these moments, the individuals could reach a break area placed in a certain radius of space from the initial position (defined at the simulation setup). Then, after the break, they will return into the classroom, at their initial position. In this occupancy process, the agent could also not maintain the initial distance conditions, especially in case of significant occupants' density in the considered building areas. The evaluation of classroom and break areas occupancy by the users is performed at given time steps, to simulate the possibility that the individuals spend a certain time near the same position during the lessons and at the lesson breaks. In this work, $\Delta t$ and $t_c$ are both assumed considering the 15 minutes threshold[1], according to Table 1, thus being equal to 1. These values are consistent with typical university buildings scheduling (e.g. breaks duration between two lessons). This choice is not aimed at representing the individuals' desires in the space fruition which generally last less than 15 minutes (e.g. motion along building paths, specific interactions among users) from a microscopic standpoint (Romero et al. 2020; Ronchi and Lovreglio 2020), but the overall effects on the whole population. Anyway, $\Delta t$ can be reduced if considering more detailed rules on pedestrians' motion (Romero et al. 2020), while the general model is maintained.

In view of the above, the adopted quick-to-apply probabilistic approach to the occupancy of an "*attraction area*" (which is shared with statistics-based SI models) ensures that each simulated agent can randomly interact with his/her neighbors (by chance) according to the proximity-based rules for the contagion spreading, in both classroom and break areas, as well as in transition periods (motion from and to these spaces). Thus, this occupancy-based approach to *Pvir* estimation is mainly affected by the occupants' density in a certain area of the *world*, rather than on motion-related issues. The *world* area *A* [$m^2$] is estimated as the sum of "*attraction areas*", by using a patches-based representation. Each patch is equal to 1 m to consider a 1:1 scaled representation of the building. The overall world area is codified according to a simplified squared area, with each side equal to $A^{1/2}$. By this way, it is possible to quickly recreate a unique layer for the whole *world*, to focus on the relationships between the individuals during the space occupancy. In this sense, since ventilation effects and individuals' motion are not directly introduced in the quick-to-apply simulation approach, the model is not actually provided with a specific representation of the space layout, such as introducing walls or other obstacles which can also influence the pedestrian motion (Romero et al. 2020). Finally, the implemented model can allow agents to freely occupy or not the *world* space. In this second case (adopted for the case study), after the lesson break, the agent returns at the previous starting position in the *world* (e.g. within the initial classroom, and so within the same group of students). This essentially allows considering the agents' use of building spaces depending on the effective building schedule.

## 2.2 Model implementation and calibration

The model described in Section 2.1 is implemented in simulation software through NetLogo (Wilensky 1999). An R script (R version 3.6.3[3]) is implemented to launch a series of simulation within the model according to previous research approaches on epidemiologic researches (Banos et al. 2015). This action ensures the application of statistical methods and the related reproducibility of scenarios in the calibration and application phases. Simulation runs were performed using NLRX package of "R statistics" programming language (Salecker et al. 2019), by defining the specific "experiment" conditions according to a series of input data.

A preliminary simulation phase verified that no influence on the final result is provided by the initial distance among

[3] https://cran.r-project.org/bin/windows/base/ (last access: 17/4/2020)

the simulated agents, as defined in Section 2.1. Then, the simulation model is calibrated to minimize the differences between the simulation outputs and the experimental data of the new contagion trends (analysis of the slope of the increasing part of the contagion curve) by the Diamond Princess cruise case study (Mizumoto and Chowell 2020). Although differences between the cruise and the university building exist, either the closed environments are "close" *worlds* characterized by the presence of the same daily users, which can perform their activities in the built environment depending on scheduled activities in it. In this sense, the *formcoeff* calibration action is aimed at trying to define an experimental-based value for the contagion speed, rather than performing arbitrary assumptions on it. For instance, if *formcoeff* = 2, the process will ideally be twice faster than for *formcoeff* = 1 (default condition in the general model). The model parameters in the calibration activities are set up by considering: (a) only *formcoeff* as variable, ranging from 0.1 to 2 (by step of 0.1); (b) constant values for the other parameters, as described in Supplementary Material S1, which is in the Electronic Supplementary Material (ESM) in the online version of this paper. 100 seeds are assigned for each experiment. The number of infected people [pp] per simulation day is evaluated as main comparison output for each sample in the test, and then related average and median values are calculated to be compared to the experimental curve. In particular, the selected *formcoeff* is the one that minimizes the average percentage difference between the infected people assessed by the model median values and the experimental values (Bernardini et al. 2020).

The Diamond Princess cruise[4] is organized in 12 decks open to the passengers, with an overall length of about 290 m, and calculated *A* of about 73500 m$^2$. During the COVID-19 emergency onboard, the cruise host 3711 individuals. The index case was embarked on the 20$^{th}$ of January 2020. On the 30$^{th}$ of January, a total of 2 observed cases were reported within the median incubation period (Lauer et al. 2020), and the index case was confirmed on the 1$^{st}$ of February 2020. In this study, we consider the increase in observed cases from this day to the maximum daily values in "new" infected people (7$^{th}$ of February), according to the observed data from previous work on the cruise event (Mizumoto and Chowell 2020). According to the general schedule provided by previous works (Fang et al. 2020), it is assumed that the occupants could spend 15 hours in contact one to each other. Since the model is based on 15 minutes-long simulation steps, 60 simulation steps are equal to 1 day. No simulation steps are performed during the night-time. Besides, it is considered that the individuals could freely move from one part of the cruise to another, by moving for about 50 m per step, one at one hour. In view of the above, the simulation lasts 420 steps, which correspond to 7 days.

[4] All the data are derived by analysing plan schemes reported at https://www.princess.com/ships-and-experience/ships/di-diamond-princess/ (last access: 6/4/2020)

## 2.3 Application to the case study

The main building in the Faculty of Engineering campus at Università Politecnica delle Marche, Ancona (Italy) has been selected as a significant case study in the university building context. It hosts about 5000 students and professors, who usually attend lessons in classrooms from Monday to Friday, for an overall lessons time of 8 hours per day.

Occupancy issues are characterized by the following main aspects:

- students are divided into course groups, and each group attends the same lessons in different classrooms during the day, thus having the possibility to move from a classroom to another.
- each lesson lasts at least 45 minutes to 1 hour. Afterwards, a lesson break is generally performed, and students generally move towards break areas.
- as a result, contagion spreading connected to occupancy areas considers how classrooms are characterized by main contacts between the same students in a course group, while break areas are affected by possible contacts between different course groups.

Since each students' group is generally considered to attend lessons in the same building area (ideally, the same floor) according to the current scheduling approach, this work does not model the whole main university building, but only a significant part of it (i.e. a single floor of classrooms and breaks areas).

Input variables parameters for the case study simulation are shown in Table 2, described by Probability Density Functions (PDFs). In particular, the maximum number of initial people *N* has been defined considering the maximum capacity of the classrooms, which corresponds to about 1 pp/m$^2$ inside the classroom. The minimum values consider previously available data of the university spaces occupancy under different scenarios (i.e. lessons, exams). *Initial infectors %* are defined by considering as minimum value the input data for the contagion in the Diamond Princess case study (compare

**Table 2** Parameters characterization for university building application

| Parameter | Min | Max | PDFs |
|---|---|---|---|
| *N* | 250 | 1150 | Uniform |
| *Initial infectors %* | 0.0546 | 30 | Uniform |
| *Mask wearing %* | 1 | 100 | Uniform |
| $prot_i$, $prot_j$ (*mask filter*) | 0 | 1 | Uniform |
| *Traveled distance* | 1 | 100 | Uniform |

to Section 2.2). A maximum value of 30% of the population is arbitrarily chosen to recreate a possible scenario for local cluster conditions in COVID-19 emergency. *Mask wearing %* has been defined as a uniform probability density function. Finally, *mask filter* has been defined even as a uniform probability density function, considering that the classification of the masks in groups (i.e. FFPk, KN) is depending on the ability to stop a specific fraction [0–1] of the aerosol drops, which can affect direct and indirect virus spreading in the adopted proximity-based model.

Constant parameters are shown in Supplementary Material S2. In particular, the epidemiological parameters are scaled according to the calibration model input set, the occupants' movement parameters are provided according to the case study configuration and the *formcoeff* is the best-fitting one according to calibration results from Section 2.2 activities.

The overall simulated time is equal to 14 days, which corresponds to 10 days of university opening (320 steps according to Supplementary Material S2). This value can represent a good estimation of the maximum incubation time from previous researches and consolidated data from international health organizations (Lauer et al. 2020)[1], leading to maximum contagion spread effects due to the initial infected people.

A Sensitivity Analysis is firstly performed on the university building scenario to understand which are the main independent variables affecting the final results (see Section 2.3.1). Then, the same simulation outputs are compared together depending on such SA results (see Section 2.3.2).

### *2.3.1 Sensitivity analysis*

The Sensitivity Analysis is performed through variance-based decomposition (Sobol' 2001). The Sobol method is used to calculate Sobol Total index (STi) and first-order sensitivity index (SFi), for any stochastic input of the performed calculation. STi measures the contribution to the output variance due to each input, including all variance caused by its interactions with any other input variables (Saltelli et al. 2007, 2010). The higher the value of the sensitivity indices, the most influential the respective input on the outcome. SFi measures indicate the main contribution of each input factor to the variance of the output. Runs are performed using NLRX package and adopting the Sobol variance decomposition scheme proposed by Saltelli et al. (2007, 2010). After some preliminary tests necessary to improve the accuracy of the proposed model, two sets of 77000 runs are performed.

### *2.3.2 Criteria for effectiveness evaluation of mitigation strategies*

The results from the simulation scenarios runs performed for Section 2.3.1 are organized depending on the following main independent solutions in contagion spreading reduction to mainly outline different conditions in building operation (Fang et al. 2020; Yang et al. 2020; Zhai 2020):

1. effect of *mask filter* as individuals' protection solution, combined with the *mask wearing %* (classified in homogeneous classes with steps of 10%), that represents the implementation level for the solution. The multiplication between *mask filter* and *mask wearing %* is introduced to quickly evaluate these parameters in a combined manner.
2. *N* as occupancy factors affecting the possibility to implement social distancing solutions. *N* values can be classified according to occupants' density *Docc* [pp/m$^2$] in classrooms, which are the occupancy areas. Lower *N*, lower the occupants' density, higher the possibility for the building stakeholders to set up social-distancing solutions inside the world. *Docc* values are offered by 0.1 pp/m$^2$ wide classes. Discretization in 4 density classes is also provide to discuss the effects in relation to the average dimension of the seats in the classrooms: (a) $250 \le N \le 350$, 0.2 pp/m$^2$ $\le$ *Docc* $\le$ 0.3 pp/m$^2$; (b) $350 < N \le 600$, 0.3 pp/m$^2$ $<$ *Docc* $\le$ 0.5 pp/m$^2$; (c) $600 < N \le 1000$, 0.5 pp/m$^2$ $<$ *Docc* $\le$ 0.7 pp/m$^2$; (d) $1000 < N \le 1150$, 0.7 pp/m$^2$ $<$ *Docc* $\le$ 1.0 pp/m$^2$.
3. *initial infector %* as access control-related factor at the initial simulation step. It is assumed that a good access control solution will detect at least 95% of the infectors while entering the building starting from the first simulation step. Thus, it is assumed that the implementation of access control strategies will imply *initial infector %* ≤5%; higher values will correspond to no implementation of access control strategies.

As an output, the final infected people percentage *dI* [%] is assessed according to Eq. (5):

$$dI = \left[1 - \frac{S_{\mathrm{f}}}{S_{\mathrm{init}}}\right]\% \tag{5}$$

where $S_{\mathrm{f}}$ [pp] is the final number of susceptible people (not infected) and $S_{\mathrm{init}}$ [pp] is the initial number of susceptible people (not initially infected). The *dI* trend expresses the contagion spreading within susceptible individuals. When *dI* tends to 0, all the individuals tend to be not infected while, when *dI* tends to 100%, all the individuals tend to be infected. Solutions effectiveness increases if *dI* is minimized. *dI* values are evaluated at the final simulation step for each considered condition in the input values, and the distributions of these values are evidenced in respect to the aforementioned input values combinations. Distribution percentiles are traced and two acceptability thresholds for the solution effectiveness are selected:

1. *dI* = 5%, which implies that at most the 5% of the population will be affected by the virus. This is a conservative threshold for the solution effectiveness estimation.

2. *dI* = 25%, which implies that at most the 25% of the population will be affected by the virus, according to a quartile analysis of the sample.

Finally, the influence of *mask filter*, *mask wearing %* and *Docc* are combined to provide a model concerning the acceptability thresholds given by *dI*. The model synthetically traces the overall effects of the combination between the aforementioned solutions. To trace such correlation, additional simulation in the range *Docc* = 0 to 0.2 pp/m$^2$ are performed to represent all the density conditions (also the value under the minimum *N* value considered as acceptable for the university building use).

## 3 Results

### 3.1 Model calibration: results

Figure 2 resumes the trend in the number of infected people in the Diamond Princess scenario simulation, by outlining average simulation data for different *formcoeff* values in respect to the experimental data of Mizumoto and Chowell (2020). Infected people are represented by a logarithmic scale. The best contagion trend seems to be related to *formcoeff* = 0.5, which additionally allows maintaining a conservative approach in infected people's estimation since it is slightly over the experimental curve. As also shown by Supplementary Material S3, the experimental data are placed close to the median values for *formcoeff* = 0.5, being the average percentage error in predictions equal to about 4% in respect to the 50$^{th}$ percentile values (12% in absolute error terms)[5]. Possible differences in the contagion spreading curve are due to specific issues in the cruise use by passengers (e.g. infectors could not "die" in the real scenario; differences in motion schedule).

Nevertheless, results confirm the capabilities of the model in defining different contagion trends according to the setup of the *formcoeff* parameter. In fact, higher the *formcoeff*, obviously higher the final number of the infected people. Furthermore, for *formcoeff* up to 0.2 (dotted lines in Figure 2), the contagion is characterized by an initial peak, and then it appears quite stable, thus limiting the effects of the virus spreading but underlining long-term effects (over the 420 simulation step limit). On the contrary, for *formcoeff* from 1.5 to 2 (continuous grey lines in Figure 2), the contagion curves have a higher peak in respect to the experimental curve, thus representing, for instance, a rapid process in which additional spreading causes can appear (e.g. poor ventilation rate, surface contacts). As a consequence, such values can amplify the short-terms effects of the contagion (e.g. for *formcoeff* = 2, the peak appears at about 360 steps).

[5] Tests related to 50 patches offer similar outcomes: average percentage error equal to 11%, and in absolute terms equal to 15%.

### 3.2 Application to the case study: results

According to the results in Section 3.1, all the simulations in the case study are performed by considering *formcoeff* = 0.5. In the following sections, the SA results are firstly discussed (Section 3.2.1) and the influence of parameters in the case study are offered (Section 3.2.2).

#### 3.2.1 Sensitivity analysis and robustness check

Table 3 resumes the total order sensitivity indices (STi) and first-order sensitivity indices (SFi). Considering STi, the Sensitivity Analysis suggests that the main source of results' uncertainty is *N*. Considering that spaces where people can move are the same for all the simulations, this value represents the occupants' density in the space and so the effect of a possible "social distancing" measures. The second source of uncertainty of results is the *mask filter*, showing also the importance of individual protection measures. The other secondary sources of results' uncertainty are *Initial infectors %* and *mask wearing %* previously defined. The effect of *traveled distance* appears negligible. Considering that the sum of SFi is less than 1 (0.94), the model is non-additive, with limited interactions between input factor, as suggested by Saltelli et al. (2007).

#### 3.2.2 Simulation scenarios results

According to the Sensitivity Analysis results, the main independent variables are combined to describe different scenarios in the case study. Thus, the effect of the *traveled distance* variable is not here discussed.

Figure 3 shows the *dI* distribution according to a boxplot representation (no outliers) depending on *Docc* classes (for each panel) and the masks implementation, described according to *mask filter*mask wearing%* classes, regardless of access control-related solutions. In general terms, the use of respiratory protective devices with higher *mask filter* values can effectively reduce the virus spreading, especially when the solution is implemented for higher *mask wearing%* values. This result is also confirmed by the general trend representation of Supplementary Material S4, which considers the different *mask filter*mask wearing%* classes regardless of density-related and access control-related solutions. Comparing the different panels of Figure 3, as well as Supplementary Material S4, it is worth noticing that at least 90% of the occupants should at least wear FFP1 masks (or more protective devices) to obtain *dI*<25% in each density conditions. The two extreme cases of *Docc* conditions concerning minimum (Figure 3(a)) and maximum (Figure 3(d))

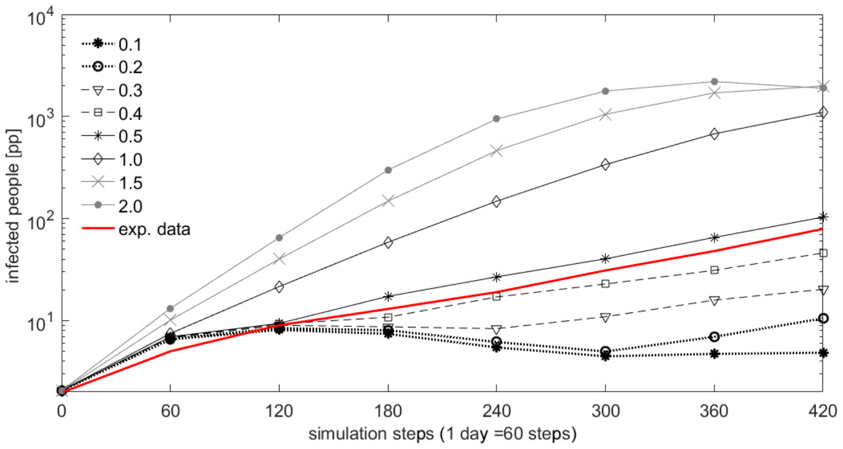

**Fig. 2** Comparison between the experimental data from the Diamond Princess cruise (red line) and the simulation results, for the main formcoeff values according to their trend. Infected people are represented by the logarithmic scale

**Table 3** Total order sensitivity indices (STi) and the first-order sensitivity indices (SFi) for the considered parameters

| Parameter | *N* | *Initial infectors %* | *Mask wearing %* | *Mask filter* | *Traveled distance* |
|---|---|---|---|---|---|
| STi | 0.796 | 0.045 | 0.038 | 0.066 | — |
| SFi | 0.871 | 0.072 | 0.073 | 0.118 | 0.006 |

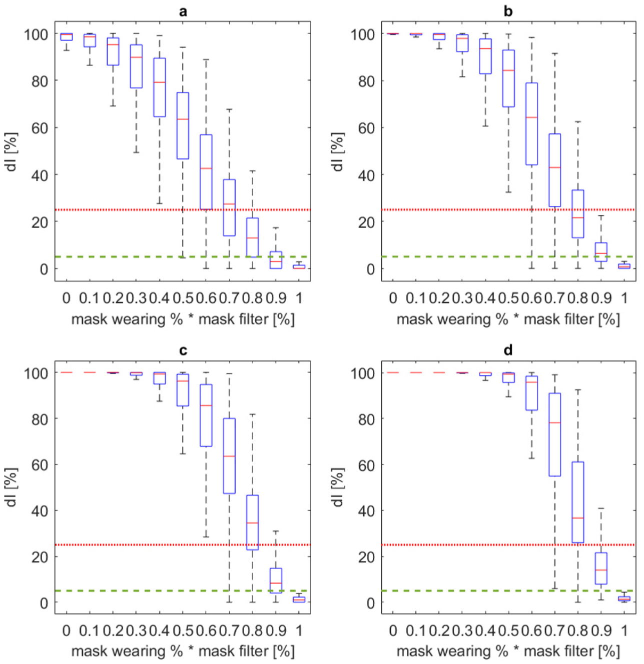

**Fig. 3** Boxplot *dI* values distribution at the last simulation step for the whole sample, with respect to the effects of different density classes: (a) $Docc \le 0.3$ pp/m$^2$; (b) 0.3 pp/m$^2$ < $Docc \le 0.5$ pp/m$^2$; (c) 0.5 pp/m$^2$ < $Docc \le 0.7$ pp/m$^2$; (d) 0.7 pp/m$^2$ < $Docc \le 1.0$ pp/m$^2$ . Values are traced according to the overall mask effect. *dI* acceptable thresholds are defined at *dI* = 5% (dashed green line) and 25% (continuous red line)

building capacity classes are evaluated in the following.

Considering the *dI* = 25% acceptability threshold, the limitation of building use to $Docc \le 0.3$ pp/m$^2$ (Figure 3(a)) allows about a 20% reduction in needed *mask filter*mask wearing%* classes in respect to density maximization conditions (Figure 3(d)). Such an outcome confirms how the use of occupants' density control strategies seems to limitedly involve acceptable solutions in terms of *dI* values if applied by themselves. To this end, please also compare Supplementary Material S5. Meanwhile, the effective possibility to engage users in maintaining social distancing during the whole occupancy timing influences the overall *dI* trend. This main reason seems to affect the width of boxplot ranges, especially in $Docc \le 0.3$ pp/m$^2$ (see Figure 3(a)) and in intermediate conditions in terms of *mask filter*mask wearing%* classes in all the Figure 3 panels.

Concerning the maximum building capacity conditions (0.7 pp/m$^2$ < $Docc \le 1.0$ pp/m$^2$), Figure 4 traces, in the different panels, the effects of *mask filter* classes on *dI*, depending on the level of implementation within the hosted population (*mask wearing %*), regardless of the access control strategies. The implementation of FFP3 masks for more than 90% of the occupants leads to *dI* values under the *dI* thresholds, as shown by Figure 4(a).

On the contrary, the adoption of surgical masks (Figure 4(c)) and non-standards protections (Figure 4(d)) by the users do not seem to allow considerably reaching the dI thresholds. These results show that a minimum protection level in terms of *mask filter* should be always guaranteed to the occupants to have limit conditions within the *dI* acceptability thresholds.

Figure 5 graphically compares the implementation of access control strategies with respect to the *mask filter*mask*

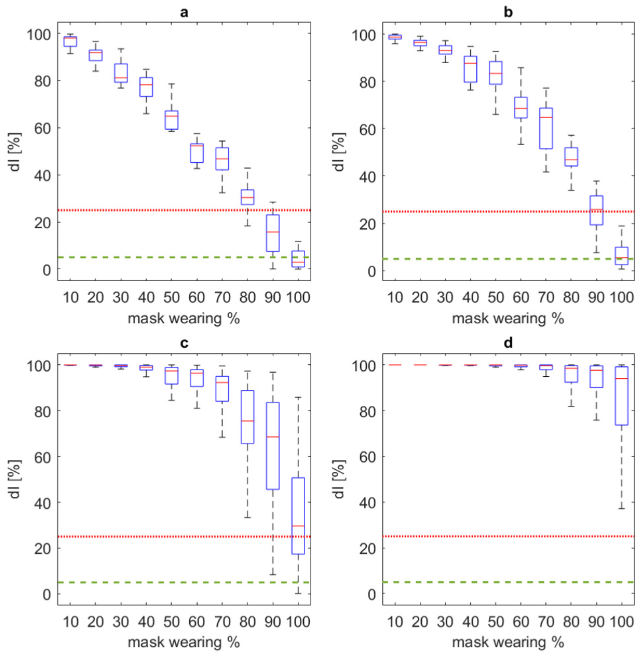

**Fig. 4** Boxplot *dI* values distribution at the last simulation step for maximum building capacity, in respect to the effects of different *mask filter* classes: (a) FFP3; (b) FFP2; (c) FFP1; (d) surgical mask. The boxplot representation is offered by distinguishing the different *mask wearing %* classes. dI acceptable thresholds are defined at *dI* = 5% (dashed green line) and 25% (continuous red line)

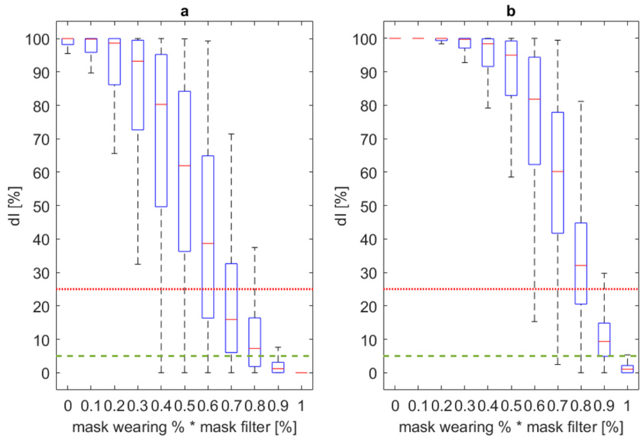

**Fig. 5** Boxplot *dI* values distribution at the last simulation step for the whole sample, with respect to: (a) access control strategies implemented and (b) no access control strategies implemented. Values are traced according to the overall mask effect. *dI* acceptable thresholds are defined at *dI* = 5% (dashed green line) and 25% (continuous red line)

*wearing%* classes, regardless of the *Docc* classes. From a general perspective, to obtain the outcoming median of *dI* < 5%, the access control strategies should be supported by more consistent solutions in terms of respiratory protective (higher *mask filter***mask wearing%* values), as expected. Anyway, it is worthy of notice that the access control (Figure 5(a)) allows a significant *dI* reduction for the intermediate range of *mask filter***mask wearing%* classes in respect to conditions in which no access control are performed (Figure 5(b)). Differences in average *dI* are limitedly reached for access control strategies implemented if considering the highest mask filter-based solutions, such as FFP3 and FFP2 implementation, even if at the maximum *Docc* values, as shown by Supplementary Material S6. On the contrary, for such mask conditions, implementing access control strategies can reduce the overall dispersion of *dI* data, especially for the class *mask wearing%***mask filter* = 80% (see Supplementary Material S6). In this condition, the *dI* interval 25$^{th}$–75$^{th}$ percentiles is −5% dispersed in respect to the same class for no access control strategies conditions. Nevertheless, poorer mask filter based solutions can take advantages of the access control strategies implementation, especially at the maximum density conditions, as traced by Supplementary Materials S7. In particular, using FFP1 by about the 100% of the population in access control conditions could lead at *dI* < 25% for more than the 50% of the simulated cases, while extreme cases for surgical mask implementation falls under the *dI* thresholds.

Finally, in view of the above, Figure 6 traces the *dI* results according to boxplot distributions, depending on the *Docc* levels, in typical easy-to-implement and recurring conditions for public buildings, because: (1) surgical masks are used by occupants, thus guaranteeing the possibility to have a

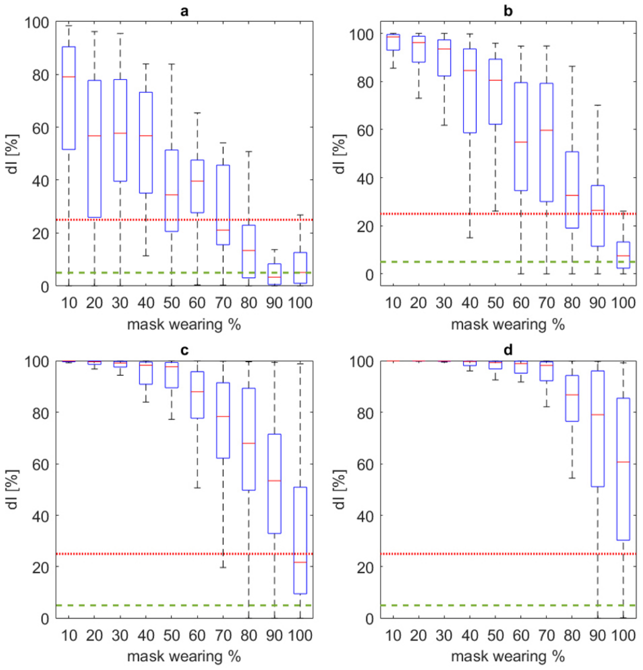

**Fig. 6** Boxplot *dI* values distribution at the last simulation step for surgical mask implementation scenarios, with respect to the effects of different density classes: (a) *Docc* ≤ 0.3 pp/m$^2$; (b) 0.3 pp/m$^2$ < *Docc* ≤ 0.5 pp/m$^2$; (c) 0.5 pp/m$^2$ < *Docc* ≤ 0.7 pp/m$^2$; (d) 0.7 pp/m$^2$ < *Docc* ≤ 1.0 pp/m$^2$ . Values are traced according to the overall mask effect. *dI* acceptable thresholds are defined at *dI* = 5% (dashed green line) and 25% (continuous red line)

widespread implementation by limiting costs and maintaining minimum comfort levels for the individuals; (2) access control strategies can be easily implemented at the building entrances. A reduction of about 20% in *mask wearing%* effort could be achieved by passing from 0.5 pp/m$^2$ < *Docc* ≤ 0.7 pp/m$^2$ (Figure 6(c)) to *Docc* ≤ 0.3 pp/m$^2$ (Figure 6(a)), if considering the *dI* threshold at 25%. Anyway, *Docc* > 0.7 pp/m$^2$ conditions should be avoided, according to Figure 6(d), in view of reaching at least the *dI* = 25% acceptability thresholds by median *dI* values. At the same time, the Supplementary Material S8 compares *dI* for surgical mask implementation and depending on *Docc* and access control strategies implementation. Results remark how avoiding access control strategies implementation cannot be always accepted according to the simulation results.

## 4 Discussion

Results show the model capabilities in predicting different effects of input conditions due to the university building spaces use and to the virus spreading characterization. The model is provided for university buildings, but it could be extended to other public building intended uses.

In general terms, the effective possibility to limit the virus spreading in university buildings could be only achieved if more than 1 risk-reduction solution will be implemented, thus confirming previous works results (Dai and Zhao 2020; Ronchi and Lovreglio 2020). Simulation results agree with previous preliminary insights on the importance of widespread facial masks adoption to limit the contagion spreading (Howard et al. 2020; Zhai 2020), especially by considering limitations to the building capacity and active access control strategies to detect symptomatic individuals and prevent their access to the university spaces. If such strategies are not combined, as expected, an adequate level of contagion mitigation can be reached only using high protection respiratory devices ("invasive" masks, like FFP ones) by almost every occupant. On the contrary, the combination between access control and building capacity limitation will lead to move towards the adoption of less invasive masks, e.g. surgical mask, by the whole number of the hosted individuals, as also suggested by review studies (Howard et al. 2020). Such a scenario could be more acceptable by the final users.

From a university stakeholder's perspective, the sustainability level depends on the specific aspects involving the strategies:

1. *for mask implementation strategies by the stakeholder*: ensuring that all the students and teachers wear facial masks characterized by a specified *mask filter* for at least the considered *mask wearing %* will imply: (a) economic efforts, such as the costs for masks supplied by the stakeholder (mainly, for teachers and other university workers; for students who try enter the building without a mask); (b) and operational efforts, such as due to activities for distribution of masks to the occupants, as well as staff actions for the control of mask-wearing behaviors by the occupants. Related cost-benefit evaluations are then needed.
2. *for density control strategies*: the possibility to perform L&T activities with a reduced number of users should be considered depending on the possibility of guaranteeing "remote access" to all the others, according to the academic continuity perspective (Dohaney et al. 2020; Favale et al. 2020). This aspect should be combined to economic evaluations as well as to the possibility to effectively deploy the measures into the building (e.g. technological access control implementation; use of building staff members at the building entrances; occupants' positioning control solutions). Additional evaluations should be performed in relation to facial masks use to increase the occupants' density (also compare to previous point 1).
3. *for access control strategies*: deploying staff members or technological solutions to check the users' health state at the entrance should face: (a) both economic and operational issues; (b) the possibility of guaranteeing rapid access by the users themselves.

Results could be also used to derive simplified rules to combine the strategies and reach, at most, the acceptable *dI* value. Figure 7 traces the correlation between *mask filter* values and *Docc* that lead to final simulation results with *dI* placed under the considered acceptability threshold (5% for Figure 7(a); 25% for Figure 7(b)), regardless of the *initial infectors %*. The *mask filter* classes adopted in previous Section 3.2.2 are also shown, while the color of data refers to the related *mask wearing %* values (color bar on the right). Finally, the interpolation of maximum values is offered according to a power-based regression approach ($ax^b + c$). It is better to evidence that the provided equations just try to give a first rough quantitative measure of the upper boundary limit not to be crossed. This means that no admitted solutions are present over the curve.

The regression shows the limit values for *mask filter values* – *Docc* pairs which can lead to acceptable scenarios according to the defined threshold. Lower values of *mask filter* imply lower values in acceptable densities, while *Docc* equal to about 1 pp/m$^2$ is admitted only for FFP3 masks implementation.

As expected, maximum pairs and the related regression curves at *dI* = 5% are lower than those at *dI* = 25%. The general trend and the regression coefficient data confirms simulation results. Some extreme cases could be highlighted by the regression trend. Considering minimum *mask filter* value (i.e. on-standards protection solutions), *Docc* tends

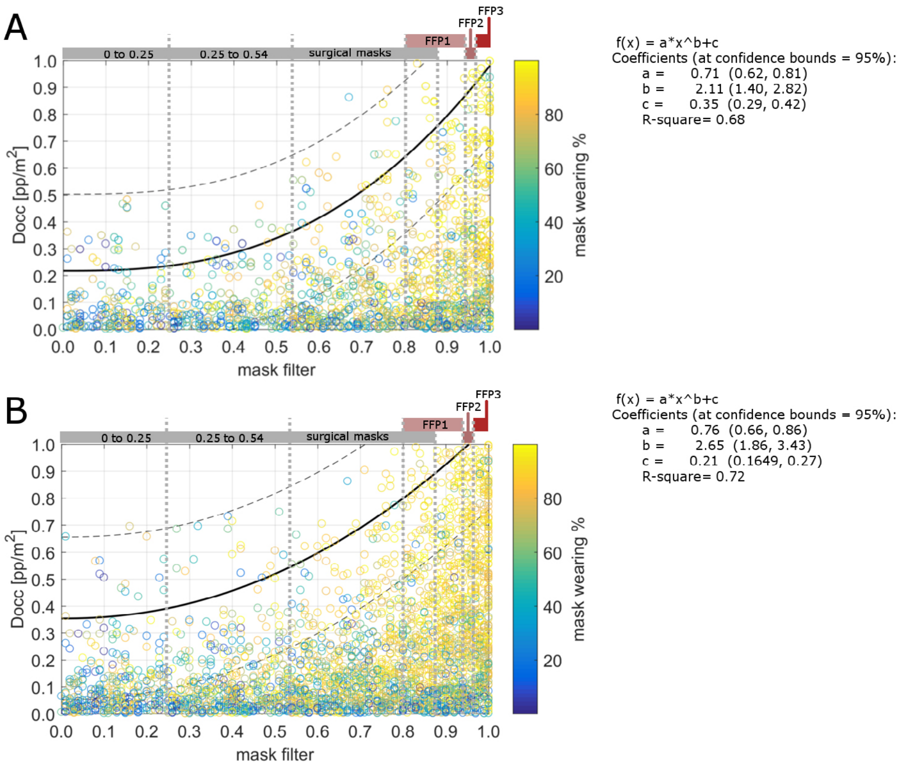

**Fig. 7** *Mask filter* – *Docc* correlation for all the pairs related to (a) $dI \leq 5\%$ and (b) $dI \leq 25\%$. The pairs' colour is related to the *mask wearing %*. Regression curves ($ax^b+c$) are shown by providing 95% of confidence intervals regression (dashed lines; see equation coefficients on the right of each panel). *Mask filter* classes are also shown in the upper part of the graphs to 0.3 pp/m$^2$ for $dI$ = 25% with access control strategies. Regression predictions move closer for FFP2 and FFP3 mask implementation. Finally, 95% of confidence intervals regression curves evidence possible upper and lower bounds of the models. In the case of a more conservative approach, in both Figure 7(a) and Figure 7(b), it should better use the lowest dashed curve (lower confidence bond at 2.5%). This curve essentially admits as an acceptable solution the adoption of surgical masks at the lowest densities (e.g., for $dI$ = 5%: for *Docc* ranging from 0.0 to 0.1 pp/m$^2$, the *mask filter* is close to 0.54).

Since the model does not include the *mask wearing %* as prediction input, the proposed regression model can be adopted also when the occupants wear respiratory protective devices with different *mask filter* values. In such conditions, it is conservatively considered that the lower *mask filter* value characterizes the scenario for the whole hosted occupants' population. Furthermore, the *mask wearing %* on Figure 7 outlines the minimum values for the pair implementation.

From a modelling perspective, results confirm how the developed simulator could effectively represent different scenarios in university buildings depending on the input factors used to estimate the virus spreading probability. The calibration of the model is performed from current available experimental data, to reproduce real-world conditions in the simulated environment. When other data will be available, it will be possible to update such calibration, by also including, for example, additional modes of person-to-person transmission means or person-to-building components (i.e., contact with contaminated objects and surfaces) or systems (e.g. ventilation systems) (Adams et al. 2016; Gao et al. 2016; Lu et al. 2016, 2020; Zhang et al. 2018; Cirrincione et al. 2020; Hu et al. 2021).

Besides, specification on the layout in terms of geometry and presence of obstacles could be implemented, to better take into account the additional architectural features affecting the virus spreading according to consolidate literature on airborne diseases (Pica and Bouvier 2012; Prussin et al. 2020). In this perspective, future modifications to the model could include the effects of path rules on pedestrians' motion inside the building spaces and the specific rooms, by also analyzing the impact of exposure in reference to specific foot traffic conditions and rules into the buildings (e.g. dedicated paths, effectiveness comparison of one-way versus two-way indoor traffic paths) (Romero et al. 2020). In this sense, the proposed modeling approach based on proximity and exposure-time criteria could be maintained by moving to smaller simulation step (i.e. lower than 15 minutes). Reducing the simulation step should be linked to more detailed rules on pedestrians' motion, while computational times will be affected too. Anyway, acceptable conditions for occupants' flows in the building in terms of needed times and safety levels against virus spreading will be then evaluated according to such future modifications.

Finally, the model considers the possibility to have "new" infected individuals only inside the building. This aspect could effectively represent most of the basic conditions in the return to "business as usual" in which the agents' life does not include the participation to social activities and limit "unsafe" contacts outside of the "working" (or "studying") places. Anyway, the daily infector percentage in the model could vary if considering additional new infected people/ infectors whose contagion happened outside of the building.

## 5 Conclusions

The COVID-19 pandemic event evidences the rule of simulation tools to support building decision-makers in evaluating the impact of reliable and feasible different risk-mitigation strategies for the safety of occupants. In fact, simulators can be used to optimize the safety measures while maintaining adequate conditions for the fruition and operation of closed environments for the stakeholder, the users and the community as a whole. In this general context, decision-makers of university buildings could take advantage of these tools in the view to avoid further academic disruption in such relevant kind of public buildings.

This paper provides the development of an agent-based model to estimate the impact of different risk-mitigation strategies against the virus spreading in university buildings. The model relies on consolidate proximity-based and exposure time-based rules given by international health organizations. A probabilistic approach is adopted to this end, by mainly focusing on the representation of occupancy issues in main areas for university Learning and Teaching (L&T) activities (i.e. classrooms and break areas), according to the related schedule, and so to the presence of the same "day-to-day" users (i.e. students and teachers). The model can also include the representation of contagion-mitigation strategies connected to the use of facial masks/respiratory protective devices, occupants' density control (in the view of maintaining social-distancing strategies) and other access control strategies. It is also able to provide a first quantitative measure of the implementation of such strategies by the relative stakeholders. After its implementation in a software tool and the calibration activities (through available experimental data in a closed environment), the simulator is applied to a significant university case study

Besides the possibility to reproduce what happened in such a COVID-19-affected closed environment, results show how the model can represent the impact of the contagion-mitigation strategies during the time. Thus, the effectiveness of each strategy (as individually adopted) and of the combination between different measures could be evaluated by the simulator.

In the considered university case study, results show that the major effectiveness seems to be reachable by using respiratory protective devices (i.e. FFPk masks), regardless of the implementation of other strategies. Nevertheless, the adoption of FFPk devices can generally imply a low level of acceptability by the occupants, because people could not feel comfortable with FFPk devices and FFPk devices are not easy-to-use devices by non-specialized users. An acceptable level of effectiveness could be reached by combining different measures, i.e. the use of facial masks and the control of occupants' densities. Limiting the number of people inside the building could support the implementation of surgical masks by the users, thus improving the mask-related operational conditions for the occupants. According to the results for the case study application, the occupants' density for users wearing surgical masks should be at most halved to reach the same effectiveness of full occupants' density with FFP3 mask worn. By this way, the effects of L&T activities disruption and limitation could be majorly avoided, but the organization of activities in a "mixed" way (presence of some users plus remote access for the others) should be encouraged. The university stakeholders could support operational decisions by the model, according to both cost-benefits and users' acceptability standpoints.

The model is a preliminary and quick-to-apply model which could increase the risk-awareness for decision-makers. Furthermore, thanks to the adopted approach (i.e. agent-based modelling techniques), the model could be easily modified to integrate different epidemiological data (including modes of virus transmission), as well as different built environment and building intended use with similar activities scheduling and occupancy tasks (e.g.: occupants' actions and presences; modification to include layout features). To this end, the probabilistic approach can support the definition of general outcomes based on the different input scenario which can appear in public building spaces, thanking the simulation-based statistics. In this sense, such future activities will move towards the application to different relevant contexts characterized by long-lasting occupants' presence in some main areas, such as tourist facilities, cultural buildings and so on. The model will supply decision-makers with basic data for cost-benefit evaluation criteria in such kinds of public buildings, by considering their building intended-use peculiarities.

**Electronic Supplementary Material (ESM):** supplementary material is available in the online version of this article at https://doi.org/10.1007/s12273-021-0770-2.

**Funding note:** Open access funding provided by Università Politecnica delle Marche.

**Open Access**: This article is licensed under a Creative Commons Attribution 4.0 International License, which permits use, sharing, adaptation, distribution and reproduction in any medium or format, as long as you give appropriate credit to the original author(s) and the source, provide a link to the Creative Commons licence, and indicate if changes were made.

The images or other third party material in this article are included in the article's Creative Commons licence, unless indicated otherwise in a credit line to the material. If material is not included in the article's Creative Commons licence and your intended use is not permitted by statutory regulation or exceeds the permitted use, you will need to obtain permission directly from the copyright holder.

To view a copy of this licence, visit http://creativecommons.org/licenses/by/4.0/

## Electronic Supplementary Material

A probabilistic model to evaluate the effectiveness of main solutions to COVID-19 spreading in university buildings according to proximity and time-based consolidated criteria
