# Supplementary material for: A probabilistic model to evaluate the effectiveness of main solutions to COVID-19 spreading in university buildings according to proximity and time-based consolidated criteria
Source: Build Simul. 2021 Feb 27;14(6):1795–809. doi: 10.1007/s12273-021-0770-2 (PMC7910197; doi:10.1007/s12273-021-0770-2)
Supplement: Supplementary file 1 — A probabilistic model to evaluate the effectiveness of main solutions to COVID-19 spreading in university buildings according to proximity and time-based consolidated criteria [file 12273_2021_770_MOESM1_ESM.pdf]

Electronic Supplementary Material

A probabilistic model to evaluate the effectiveness of main solutions to COVID-19 spreading in university buildings according to proximity and time-based consolidated criteria

Marco D'Orazio, Gabriele Bernardini, Enrico Quagliarini (✉)

Department of Construction, Civil Engineering and Architecture, Università Politecnica delle Marche, via Brecce Bianche 60131 Ancona, Italy

Supporting information to https://doi.org/10.1007/s12273-021-0770-2

Supplementary Material S1 Model calibration setup for the Diamond Princess cruise scenario application: parameters assumed as constant values:

Parameter	Value	Source
A	73500m^2 (271 patches per side)	The overall assessed surface area of the cruise from a graphical evaluation (based on the plans)
N	3711	Fang et al. 2020; Mizumoto et al. 2020
p_{imm}	0 %	No evidence that immune people can exist
Initial infector %	0.054%	Considering experimental data (Mizumoto and Chowell 2020), it is calculated as the ratio between 2 observed cases within the median incubation period (Lauer et al. 2020) and *N*
Asymptomatic ratio	20%	The superior limit in the confidence interval of estimated asymptomatic proportion (among all infected cases) (Mizumoto et al. 2020)
Average delay	60	Equal to 1 day to be shorter than the time to display symptoms (e.g. fever onset) by the 2.5% of infected persons (Fang et al. 2020; Lauer et al. 2020)
I_{inc}	320	Corresponding to the median incubation time (and the inferior limit of the confidence interval), according to a conservative approach. It corresponds to about 5.1 days (Lauer et al. 2020)
I_{fev}	160	The average value corresponds to the minimum time to display symptoms by the 2.5% of infected persons (Lauer et al. 2020). A standard deviation is associated to make it ranging from 0 to 320 steps
$prot_i = prot_j$ (*mask filter*)	0	It is considered that all the samples are characterized by the same protection level given by the *mask filter*, to consider uniform conditions in a conservative approach (Fang et al. 2020). No masks are worn in the calibration simulation (Fang et al. 2020; Mizumoto and Chowell 2020)
t_c	1 step = 15 minutes	Consolidated data about closed environment contagion spreading from national and international health organization
Mask wearing %	0%	No masks are worn (Fang et al. 2020; Mizumoto and Chowell 2020)
Traveled distance	20 patches; 50 patches	Two supposed distance between the different main locations in the cruise are tested

E-mail: e.quagliarini@staff.univpm.it

Supplementary Material S2 Case study application: parameters assumed as constant values:

Parameter	Value	Source
A	4300 m^2 (66 patches per side)	The overall assessed surface area of the considered part of the building, by including all the spaces accessible by the students (i.e. classrooms, spaces for study)
formcoeff	Best fitting value according to model calibration activities in Section 2.2	Deriving the spreading phenomenon according to previous experimental data in a closed environment
p_{imm}	0 %	As for the calibration test, since no evidence that immune people can exist
Asymptomatic ratio	20%	As for the calibration test
Average delay	32	Equal to 1 day with 8 hours of attendance by students, by scaling the calibration test parameters
I_{inc}	170	Scaling the calibration test parameters depending on the steps per day length
I_{fev}	87	Scaling the calibration test parameters depending on the steps per day length
t_c	1 step = 15 minutes	Consolidated data about closed environment contagion spreading from national and international health organization, compare to Section 2.1

Supplementary Material S3 Comparison between the experimental data from the Diamond Princess cruise (red line) and the simulation results, for the best formcoeff values (0.5), by considering different percentiles and mean data:

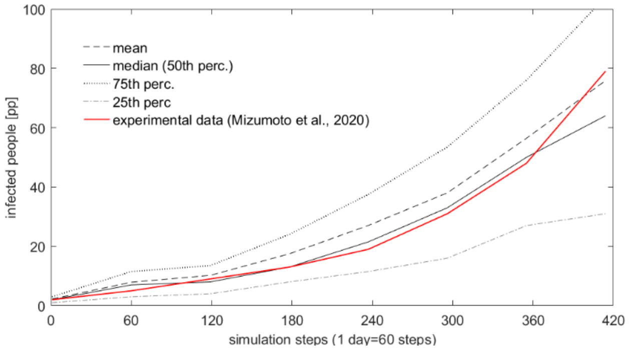

Supplementary Material S4 Boxplot *dI* values distribution at the last simulation step for the whole sample, with respect to the effects related to mask (*mask filter* and *mask wearing %*). *dI* acceptable thresholds are defined at *dI* = 5% (dashed green line) and 25% (continuous red line). Acceptability thresholds can be reached when implementing at least *mask filter*mask wearing%* ≥ 80% for *dI* = 25% and ≥ 0.90% for *dI* = 5%:

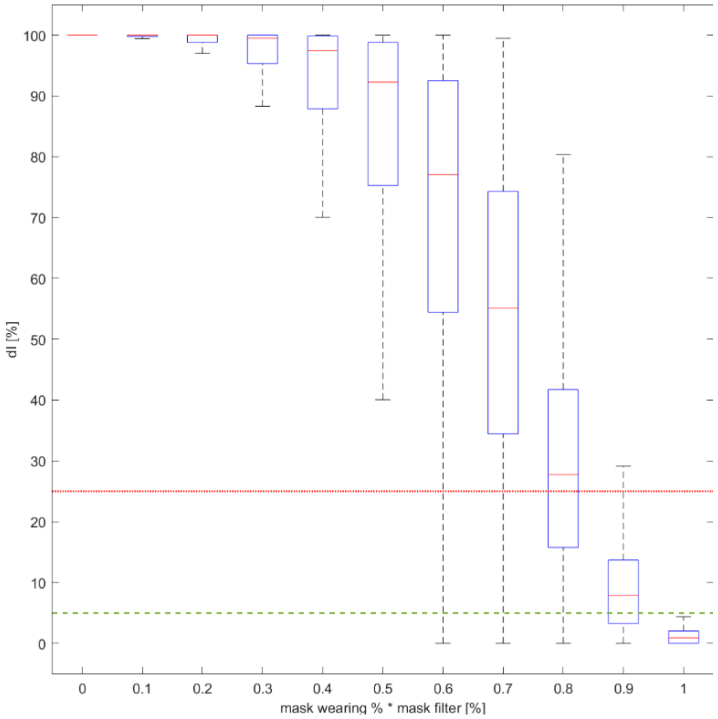

Supplementary Material S5 Boxplot *dI* values distribution at the last simulation step for the whole sample, with respect to the effect of occupants' density *Docc* values discretized by 0.1 pp/m^2. *dI* acceptable thresholds are defined at *dI* = 5% (dashed green line) and 25% (continuous red line):

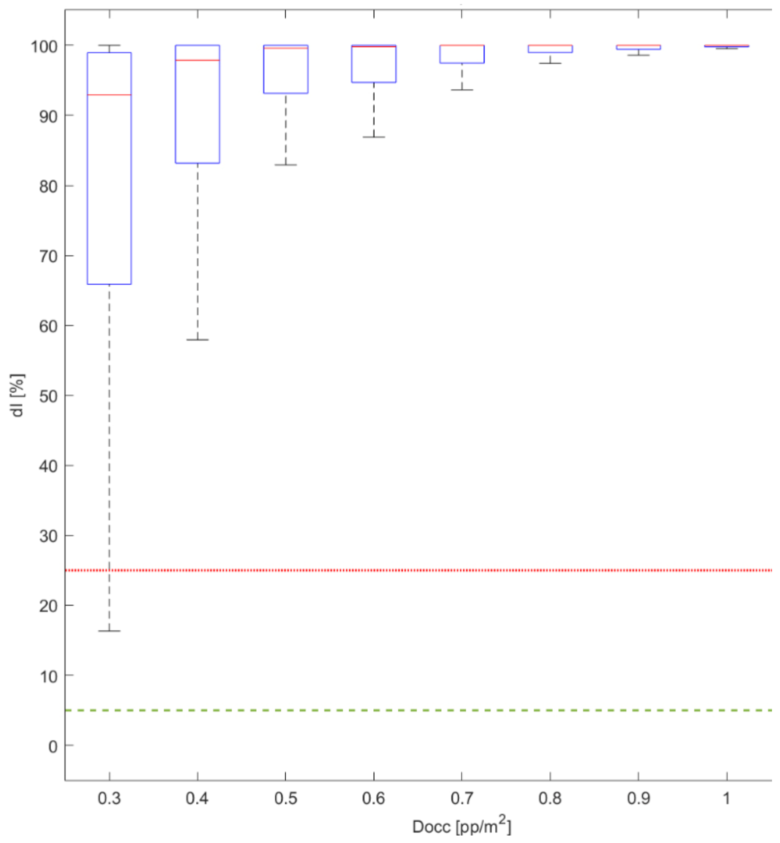

Supplementary Material S6 Boxplot *dI* values distribution at the last simulation step for maximum building capacity, in respect to: (a) no access control strategies implemented and (b) access control strategies implemented. Values are traced according to the overall mask effect. *dI* acceptable thresholds are defined at *dI* = 5% (dashed green line) and 25% (continuous red line):

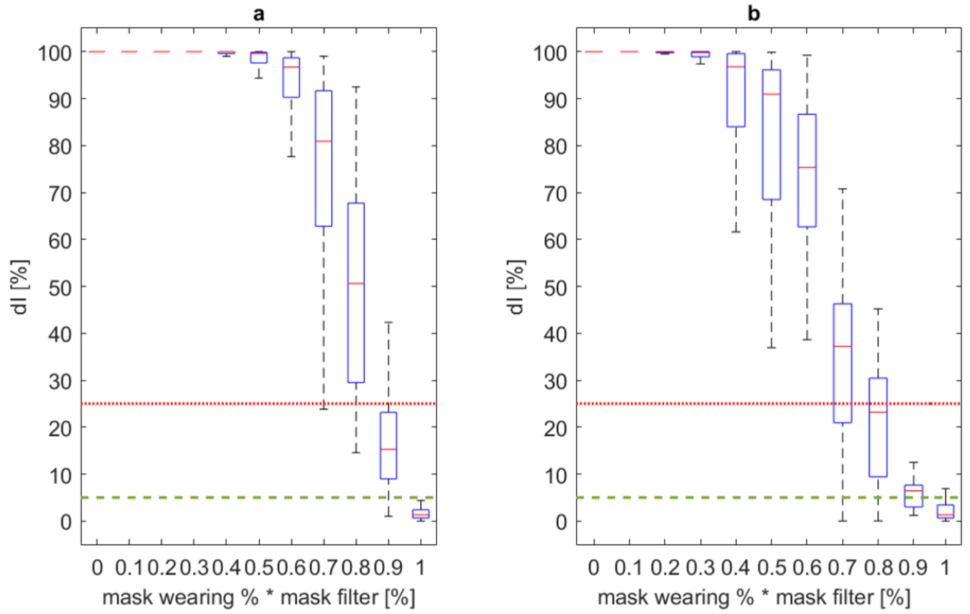

Supplementary Material S7 Boxplot *dI* values distribution at the last simulation step for maximum building capacity when access control strategies are considered, in respect to the effects of different *mask filter* classes: (a) FFP1; (b) surgical masks. The boxplot representation is offered by distinguishing the different *mask wearing %* classes. *dI* acceptable thresholds are defined at *dI* = 5% (dashed green line) and 25% (continuous red line). In particular, in panel a, the FFP1 median at *mask wearing%* = 100% is equal to about 10%:

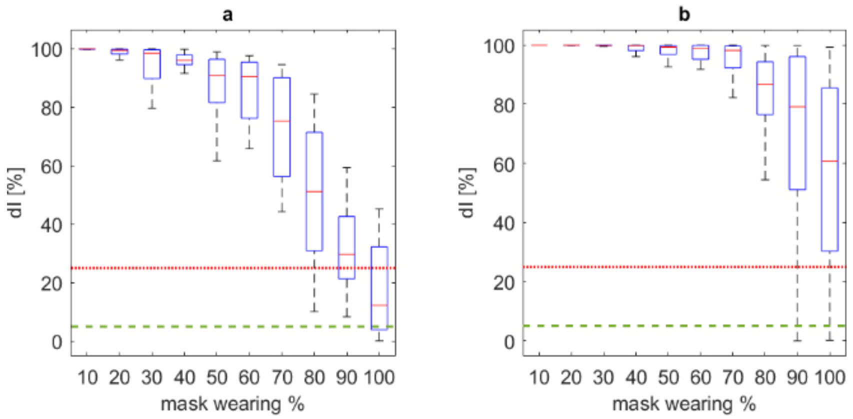

Supplementary Material S8 Boxplot *dI* values distribution at the last simulation step for surgical masks implementation in relation to the occupants' density *Docc* classes, with respect to: (a) no access control strategies implemented; (b) access control strategies implemented. Data are offered regardless of the *mask wearing %* classes. *dI* acceptable thresholds are defined at *dI* = 5% (dashed green line) and 25% (continuous red line):

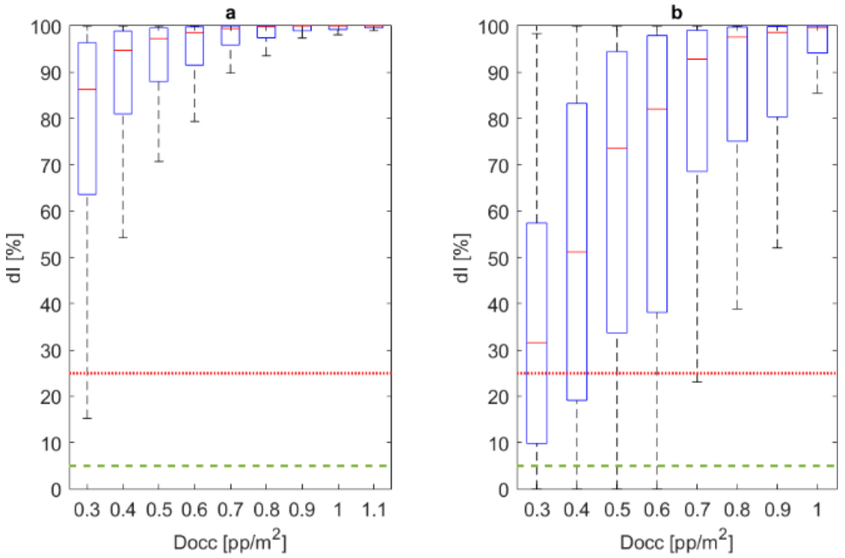

References

Fang Z, Huang Z, Li X, et al. (2020). How many infections of COVID-19 there will be in the "Diamond Princess"—Predicted by a virus transmission model based on the simulation of crowd flow. arXiv:2002.10616.

Lauer SA, Grantz KH, Bi Q, et al. (2020). The incubation period of coronavirus disease 2019 (COVID-19) from publicly reported confirmed cases: estimation and application. *Annals of Internal Medicine*, 172: 577–582.

Mizumoto K, Chowell G (2020). Transmission potential of the novel coronavirus (COVID-19) onboard the diamond Princess Cruises Ship, 2020. *Infectious Disease Modelling*, 5: 264–270.

Mizumoto K, Kagaya K, Zarebski A, et al. (2020). Estimating the asymptomatic proportion of coronavirus disease 2019 (COVID-19) cases on board the Diamond Princess cruise ship, Yokohama, Japan, 2020. *Eurosurveillance*, 25(10): 2000180.
